# Distribution of ABO and Rhesus-D blood groups in and around Bangalore

**DOI:** 10.4103/0973-6247.59391

**Published:** 2010-01

**Authors:** Sundar Periyavan, S. K. Sangeetha, P. Marimuthu, B. K. Manjunath, D. M. Seema

**Affiliations:** Department of Neuropathology, Transfusion Medicine Center, National Institute of Mental Health and Neurosciences, Bangalore, India; 1 Department of Biostatistics, Transfusion Medicine Center, National Institute of Mental Health and Neurosciences, Bangalore, India

Sir,

The frequencies of ABO and Rhesus-D blood groups vary from one population to another. There are no data available for Bangalore, Karnataka. Our aim was to determine the distribution of different blood groups in this region. Blood group determination was carried out for 8 years, from January 2000 to December 2007, and encompassed 36,964 subjects donating blood to the transfusion medicine center of a neurological tertiary care institute.

ABO and Rh blood grouping was done by using commercially available anti-sera A, B, AB, H and Rh (D), and known cells prepared, in-house, from pooled blood units, were used. For typing of Rh, we did not use other anti-sera like anti-c, anti-C, anti-e, anti-E; but only anti-D, which is most immunogenic. Hence those who tested positive with anti-sera D were considered to be Rh positive and those who did not were considered to be Rh negative. These anti-sera were validated at our laboratories before using them. For determination of ABO blood groups, both forward and reverse groupings were carried out.

The results were analyzed and data compiled. Our study involving 36,964 donors, both male and female, showed O group to be high, viz., 14,716 (39.81%) donors, followed by B group having 11,071 (29.95%) donors and A at 8,817 (23.85%) and AB at 2,358 (6.37%) donors being the lowest. Rh-D blood group frequency was 94.20% positive and 5.79% negative, which shows that it follows the Asiatic trend of O > B > A > AB. There were only 2 (0.005%) donors out of 36,964 with Bombay blood group (O_h_).

Few studies of ABO and Rh blood group prevalence among the various populations of India have been carried out. Study done by Nanu and Thapliyal in the north Indian population report that group B is the most predominant one,[1] as also reported in a study in neighboring Pakistan.[1,2] The south Indian study by Das *et al.* shows that group O is the most predominant one, followed by group B and group A, which is in agreement with our study; and also, the finding regarding Rh negativity was almost similar to that from our study.[3] Another south Indian study conducted on the population of Chittoor district of Andhra Pradesh also showed similar pattern of distribution of blood groups.[4]

It is hoped that the data generated in this study would assist in the planning and establishment of a functional blood service that would meet the ever-increasing demand for safe blood and blood products.
